# Adaptive assessment based on fractional CBCT images for cervical cancer

**DOI:** 10.1002/acm2.14462

**Published:** 2024-07-27

**Authors:** Yankui Chang, Yongguang Liang, Haotian Wu, Lingyan Li, Bo Yang, Lipeng Jiang, Qiang Ren, Xi Pei

**Affiliations:** ^1^ School of Nuclear Science and Technology University of Science and Technology of China Hefei China; ^2^ Department of Radiation Oncology Chinese Academy of Medical Sciences, Peking Union Medical College Hospital Beijing China; ^3^ Anhui Wisdom Technology Company Limited Hefei China; ^4^ Department of Radiation Oncology First Affiliated Hospital of Jinzhou Medical University Shenyang China

**Keywords:** adaptive assessment, adaptive radiotherapy, artificial intelligence, fractional CBCT images, Monte Carlo based dose calculation

## Abstract

**Purpose:**

Anatomical and other changes during radiotherapy will cause inaccuracy of dose distributions, therefore the expectation for online adaptive radiation therapy (ART) is high in effectively reducing uncertainties due to intra‐variation. However, ART requires extensive time and effort. This study investigated an adaptive assessment workflow based on fractional cone‐beam computed tomography (CBCT) images.

**Methods:**

Image registration, synthetic CT (sCT) generation, auto‐segmentation, and dose calculation were implemented and integrated into ArcherQA Adaptive Check. The rigid registration was based on ITK open source. The deformable image registration (DIR) method was based on a 3D multistage registration network, and the sCT generation method was performed based on a 2D cycle‐consistent adversarial network (CycleGAN). The auto‐segmentation of organs at risk (OARs) on sCT images was finished by a deep learning‐based auto‐segmentation software, DeepViewer. The contours of targets were obtained by the structure‐guided registration. Finally, the dose calculation was based on a GPU‐based Monte Carlo (MC) dose code, ArcherQA.

**Results:**

The dice similarity coefficient (DSCs) were over 0.86 for target volumes and over 0.79 for OARs. The gamma pass rate of ArcherQA versus Eclipse treatment planning system was more than 99% at the 2%/2 mm criterion with a low‐dose threshold of 10%. The time for the whole process was less than 3 min. The dosimetric results of ArcherQA Adaptive Check were consistent with the Ethos scheduled plan, which can effectively identify the fractions that need the implementation of the Ethos adaptive plan.

**Conclusion:**

This study integrated AI‐based technologies and GPU‐based MC technology to evaluate the dose distributions using fractional CBCT images, demonstrating remarkably high efficiency and precision to support future ART processes.

## 1 INTRODUCTION

Cervical cancer is one of the most common malignant tumors, ranking first in the incidence of gynecological malignancies.^1^, ^2^ Radiation therapy is a useful treatment method for cervical cancer. In the traditional radiotherapy process, physicians and physicists develop treatment plans based on the initial computed tomography (CT) images of the patient, which are used for all subsequent fractionated radiotherapy treatments.^3^ However, the patient's daily anatomical changes can cause the structural inconsistency between the original planning CT images and fractional images,^4^ resulting in inaccurate radiation dose transmission and even treatment failure. Yan et al.^5^ proposed the adaptive radiotherapy (ART) strategy that makes timely adjustments to the treatment plans in response to such anatomical changes. Currently, the ART guided by different modal images as fractional images have developed rapidly,^6^, ^7^, ^8^, ^9^, ^10^ where CBCT‐guided online ART could significantly shorten the total time for patients to maintain a fixed position with good pelvic soft tissue display resolution and shows an enormous advantage in cervical cancer.^8^

Online ART is the process of adjusting treatment plan before receiving radiation, which requires the integration of multiple steps, including the rapid delineation of target and organs at risk, the regeneration of the treatment plan, quality assurance of the plan, and the transmission of the plan. The entire process lasts from 20 to 60 min. Benefiting from the consistent isocenter points of CBCT image scanning equipment and linear accelerator treatment machine, there is no need to move the patient after obtaining CBCT images, so CBCT‐guided online ART takes less time, around 20 min. Unfortunately, there are some unavoidable issues in online ART. Online ART requires a large amount of equipment and resources and it is unrealistic to implement online ART in countries with a large number of tumor patients. On the other hand, patients need to maintain the same posture on the couch during the entire process, the patients’ comfort level and treatment experience will be greatly reduced, and changes in position and anatomical structure will inevitably occur. For example, the volume of organs such as the bladder may change during treatment, which in turn compresses the target area, causing changes in the shape of the target area.^11^, ^12^ Therefore, the inaccurate dose transmission of treatment may still occur even if the ART process is implemented. To solve the problem, we think that if the plan evaluation can be quickly completed based on the anatomical structure of the day, it can assist in determining the necessity of ART process. For patients with small changes in anatomical structure, not implementing online adaptive plan adjustment may be a better choice, which can not only avoid waste of manpower and resources but also ensure the accuracy of radiation dose transfer.

There is a significant subjectivity in manually determining when to implement ART process, and qualitative analysis can bring significant errors, which is time‐consuming and laborious, unable to meet the needs of online ART. Sicilia et al.^13^ established a binary classification model and predicted the probability of ART for non‐small cell lung cancer patients by extracting radiomics features. Gros et al.^14^, ^15^ conducted a retrospective study on head and neck patients using the RTapp workflow to evaluate the dose received by each structure. The workflow of RTapp is mainly divided into three steps: (1) first, register the planning CT images with the current day's CBCT images to generate a deformation vector field (DVF); (2) then, the generated DVF is used to propagate the contours and dose to obtain the contour information and dose distribution based on the anatomical structure of the day; (3) finally, the dose volume histogram (DVH) and dosimetric parameters of the day can be evaluated. Nigay et al.^16^ used MIM software to evaluate offline ART for a prostate cancer patient automatically. The DVF generated by CT and CBCT image registration was used to map contour information and dose distribution, and then analyze the changes in cumulative DVH and dose.

The plan evaluation based on fractional images should be compared with the original plan to observe differences in dose transmission, analyzing the realistic dose received by the target area and organs at risk. Therefore, plan evaluation based on fractional images requires two necessary information: accurate contour information and accurate dose distribution. Gros and Nigay et al.^14^, ^16^ obtained contour information and dose distribution through DVF generated by registration of planning CT images and CBCT fractional images. Some studies^17^, ^18^ have shown that the contour obtained by DVF has poor accuracy for deformable organs (such as the bladder), In addition, some researchers have questioned and discussed the accuracy of obtaining dose distribution through DVF.^19^, ^20^, ^21^, ^22^, ^23^ Although this method can automatically obtain quantitative evaluation results on fractional images, its accuracy needs to be improved.

With the assistance of artificial intelligence technology and fast Monte Carlo (MC) calculation methods, rapid and accurate plan automatic evaluation based on fractional CBCT images has become possible. This study proposes an automatic plan evaluation method based on fractional CBCT images by re‐generating the contours and dose distributions of the day, which provides a reference for online ART. The key technologies required include image segmentation, image registration, image synthesis, and dose calculation. The clinical patient data were collected for treatment on the CBCT‐guided online adaptive treatment system Ethos to test the accuracy of the proposed method in this study.

## 2 MATERIALS AND METHODS

**FIGURE 1:**
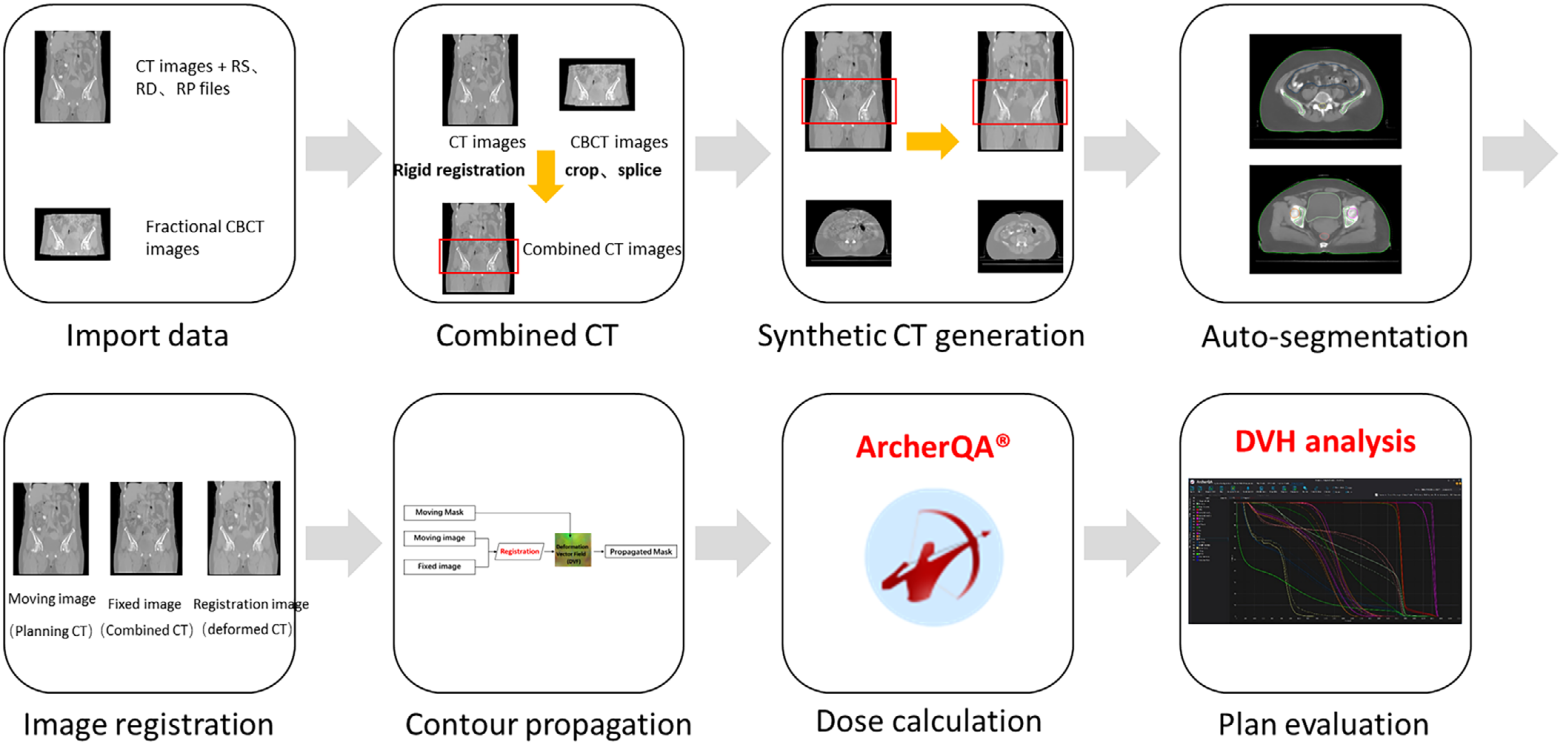
The flow chart of ArcherQA Adaptive Check.

In this study, the plan evaluation method is composed of multiple steps and methods, the flow chart is shown in Figure 1, including eight steps: data import, rigid registration of CT and CBCT images for cutting and splicing, synthetic CT (sCT) generation, auto‐segmentation based on sCT, image registration, contour propagation, dose calculation, and plan evaluation.

1. Necessary data needs to be imported to ArcherQA, including planning CT images and corresponding radiotherapy (RT) files (RT Plan, RT Dose, and RT Structure), as well as fractional CBCT images scanned before treatment on the day.
2. With the planning of CT images as the reference images, rigid registration of CBCT images and CT images is performed to obtain combined CT through cropping and splicing.
3. The CBCT part of combined CT is converted into sCT.
4. Then, auto‐segmentation of some deformable organs (small intestine, rectum, and bladder) is performed in the sCT image, and the delineation information from the original CT in areas outside the sCT is used directly.
5. The deformable image registration is performed from the original planning CT images to the combined CT images to obtain the deformed CT images and contour propagations of other organs (femoral head, bone marrow, and spinal cord).
6. After that, the obtained contours of organs are used to perform region‐of‐interest (ROI) registration to generate target contours, and the contours need to be manually modified and confirmed if necessary.
7. Subsequently, the ArcherQA dose calculation engine is performed with the deformed CT images to obtain a new dose distribution on fractional images.
8. Finally, dose evaluation is conducted based on the obtained contour information and dose distribution, which is compared with the dose in the original CT images to assist clinical doctors and physicists in determining when to use online ART.

All algorithms in this study have been integrated into the commercial verification system, ArcherQA, which is named ArcherQA Adaptive Check. The key technologies will be introduced in the following.

### 2.1 Combined CT

**FIGURE 2:**
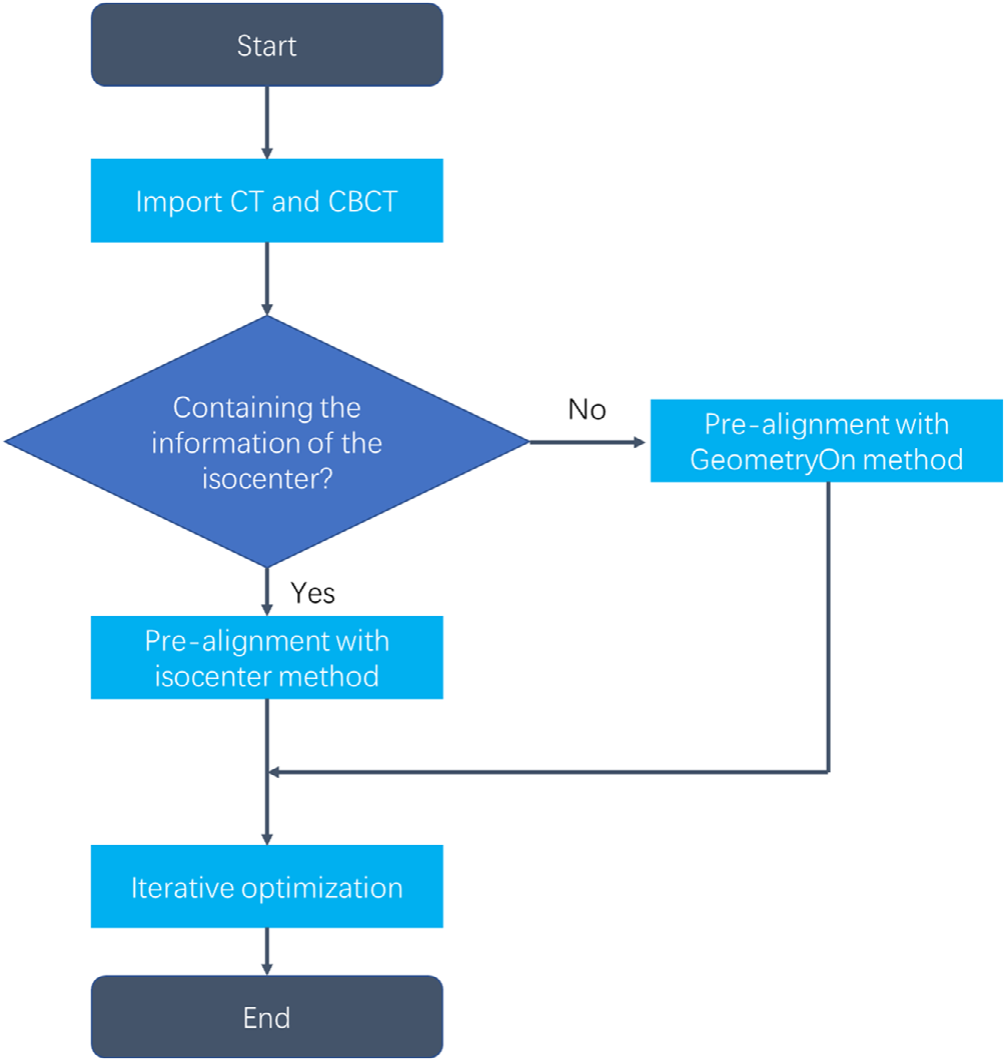
The rigid registration based on the isocenter prealignment.

The combined CT is generated from planning CT images and fractional CBCT images, where the rigid registration is the key algorithm. In this study, the rigid registration algorithm is used to align fractional CBCT images with planning CT images accurately, which is developed based on the ITK open‐source library.^24^, ^25^ The VersorRigid3DTransform model in ITK is used and the output is an array of six elements, which are the rotation angles and the translations along the *x*, *y*, and *z* axes. Before optimization, ITK have two built‐in center prealignment methods, GeometryOn and MomentsOn, which are based on the inherent information of the image and do not consider extra information. In this study, the rigid alignment is performed from CBCT images to the planning CT images. With experience in the clinic, the technician positions the patient before CBCT scanning, so the patient's irradiation center (isocenter point) is consistent with the CBCT scan center. This means that the isocenter point in the planning CT is very close to the zero point (0, 0, and 0) in the CBCT image. Therefore, this study proposes a rigid registration process based on isocenter prealignment, as shown in Figure 2. The CT and CBCT images to be registered are analyzed first. If there is an RT Plan file and the isocenter coordinates are read for center prealignment of CT and CBCT images, which is equivalent to aligning the marker lines in the clinical radiotherapy process. If there is no RT Plan file or there is no isocenter information in the RT Plan file, the GeometryOn method is used for center prealignment. After obtaining prealigned CBCT and CT images, iterative optimization is carried out to produce accurate registration results ultimately.

### 2.2 Synthetic CT generation and auto‐segmentation

Due to the poor quality of CBCT images, auto‐segmentation and dose calculation based on CBCT images pose certain challenges. Moreover, the current mainstream auto‐segmentation software is based on CT image data, and directly using CBCT images has poor accuracy. Therefore, in order to better use CBCT images in ART, it is necessary to improve the image quality of CBCT images. In this study, the CBCT images were converted to synthetic CT images, which is based on the CycleGAN network model framework^26^ with improved data normalization processing and generator model structure, shown in detail in our previous works.^17^, ^18^ Paired planning CT images and CBCT images are used to construct the model for generating synthetic CT images based on CBCT.

The auto‐segmentation algorithm is used to obtain the contours of some deformable organs, such as the bladder, rectum, and small intestine. We have done a lot of auto‐segmentation work based on CT images before and integrated it into a commercial target and organs auto‐segmentation software, DeepViewer.^27^ Therefore, the DeepViewer is used directly for auto‐segmentation of deformable organs based on model‐generated synthetic CT images.

### 2.3 Image registration and contour propagation

The deformable image registration algorithm is used to propagate the contours of some organs and obtain deformed CT images for dose calculation. This study contains two elastic registration methods, one is a cascaded network registration method based on artificial intelligence,^17^, ^18^ and the other is an elastic registration method based on B‐splines.^28^, ^29^ Artificial intelligence registration methods can quickly and accurately complete the registration process, but cannot achieve enough excellent accuracy in external datasets. The B‐spline registration method is a continuous iterative optimization process that takes a long time. Considering the differences in fractional CBCT image quality of different machines, artificial intelligence registration method is preferred when the data difference is not significant. In cases where the data difference is significant, the artificial intelligence registration method may perform poorly, and the B‐spline registration method can be used to achieve better results. The specific introduction of the two registration methods has been explained in the previous works.^17^, ^18^

In addition, we propose the structural information guided region‐of‐interest (ROI) registration, which generates the deformation field for propagating the target contours. ROIs are defined as structures that can affect the deformation of the target area, including the skin and organs at risk around the target area, which are consistent with the RT structure file to make sure that the ROIs are delineated in the planning CT images. For cervical cancer, ROIs include skin, left femoral head, right femoral head, pelvis, spinal cord, rectum, small intestine, and bladder. First, the contours of ROIs located around the target area are extracted from the planning CT and CBCT images, and assigned mask values based on the importance for the target. Therefore, the voxels within air, skin, left femoral head, right femoral head, pelvis, spinal cord, rectum, small intestine, and bladder are assigned values of 0, 1, 2, 3, 4, 5, 6, 7, and 8, respectively. Based on the mask (Mask CT) in the planning CT and the mask (Mask CBCT) in the CBCT, the deformation vector field (DVF) is optimized using the B‐spline registration method. Finally, the contours of the target in the planning CT are propagated to the CBCT based on this DVF, thereby achieving auto‐segmentation of the target in the fractional CBCT image.

### 2.4 Dose calculation

MC dose calculation is considered the gold standard for dose calculation in radiotherapy, which can fully consider the physical reactions during particle transport by simulating many particles, including lateral electronic scatter and lateral electronic disequilibrium. Our team previously developed a graphics processing unit (GPU) accelerated MC dose verification system, ArcherQA, whose accuracy has been validated in photon radiotherapy.^30^, ^31^, ^32^, ^33^, ^34^, ^35^ Therefore, ArcherQA is used to recalculate the dose according to the original RT Plan file on the anatomical structure of CBCT images to obtain the latest dose distribution. The images used for calculation are the deformed CT images or synthetic CT images, and the accuracy has been confirmed in previous studies.^17^, ^18^

### 2.5 Datasets and evaluation metrics

First, 115 cervical cancer patients treated on Halcyon machines at Peking Union Medical College Hospital were collected to test the accuracy of key algorithms, all of whom were treated with two‐course radiotherapy, 20 fractions for the first course and 8 fractions for the second course. After the first CT (CT1) scan of the patient and the formulation of radiotherapy plan (plan1), the subsequent 20 fractional treatment will be implemented according to plan1, with CBCT images scanned once before each fractional treatment. After 20 fractional treatments, the patient was scanned the second CT images (CT2), and the second RT Plan (plan2) was generated to complete the remaining 8 fractional treatments. The two‐course radiotherapy can be seen as a simplified form of offline ART. More information about the dataset was described in detail in previous works.^17^, ^18^ Therefore, each patient has two sets of CT data, Dataset1 (Paired CT1/CT2 images) was constructed. In addition, the first fractional CBCT images of patients were collected to construct Dataset2 (Paired CT/CBCT images). To evaluate the whole algorithm, 10 post‐operative cervical cancer patients who completed full process of ART on the Ethos system were collected to construct Dataset3 (ART Data). Each patient in Dataset3 had 25 fractional data, containing the fractional CBCT images, the scheduled plan (the reference plan recalculated on current anatomy) and adaptive plan (the newly optimized treatment plan on current anatomy), where the contours of target and OARs were reviewed and manually modified by the physician.

The Dice Similarity Coefficient (DSC) is used for the evaluation of contour accuracy for OARs and targets, gamma pass rate of ArcherQA versus Eclipse treatment planning system is used for the evaluation of ArcherQA dose calculation accuracy. As for the evaluation of ArcherQA Adaptive Check, the clinical interested dosimetric parameters were calculated, including the D_99_ and V_100%_ of clinical target volume (CTV), D_95_ and V_100%_ of planning target volume (PTV), V_40_ of bladder, V_40_ of rectum, V_40_ of bowel, V_10_ of bone marrow, V_30_ of left and right femoral heads. Where D_i_ means the dose received by i% of volume, V_i_ means the volume fraction of OARs irradiated by i Gy, and V_100%_ means the volume fraction of CTV and PTV receiving 100% of the prescribed dose.

## 3 RESULTS

**FIGURE 3:**
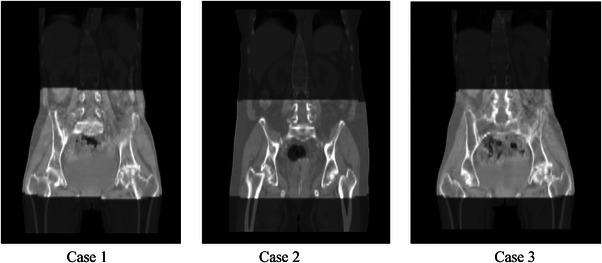
Display of rigid registration results for randomly selected three cases. The transparency is 0.5, the foreground image is CBCT and the background image is CT image. CBCT, cone‐beam computed tomography; CT, computed tomography.

The accuracy of some key technical algorithms was first verified on Dataset1 and Dataset2, respectively. Figure 3 shows the rigid registration results for randomly selected three cases. The foreground image is CBCT and background image is CT image. At the boundary of the CBCT image, the contour of the body and the continuity of the bones are excellent, the transition is natural, and there is no obvious dislocation phenomenon.

Table 1 shows the DSC values of deformed planning CT (dpCT) contours and sCT based auto‐segmentation contours for organs at risk in cervical cancer, where the contours delineated on CBCT images manually were considered as the ground truth. Overall, the accuracy of sCT‐based auto‐segmentation was higher than the accuracy of propagated dpCT contours, especially for the deformable organs (small intestine, rectum, and bladder) with significant difference (*p* < 0.001). For rigid organs (femoral head, bone marrow, and spinal cord), although the DSC of sCT‐based contours was still higher, the difference between sCT contours and dpCT contours was small compared for the deformable organs. The DSC of dpCT contours for rigid organs was about 0.9, which is acceptable according to the AAPM TG 132 report.^36^

**TABLE 1:** DSC of dpCT contours and sCT based auto‐segmentation contours for 40 paired of CT/CBCT data in Dataset2.

Contours	Small intestine	Rectum	Bladder	Femoral head L	Femoral head R	Bone marrow
dpCT	0.844 ± 0.070	0.661 ± 0.119	0.756 ± 0.125	0.917 ± 0.050	0.894 ± 0.115	0.883 ± 0.055
sCT	0.903 ± 0.054	0.789 ± 0.100	0.884 ± 0.072	0.924 ± 0.012	0.911 ± 0.064	0.922 ± 0.011
*p* ^*^‐value	*p* < 0.001	*p* < 0.001	*p* < 0.001	0.459	0.418	*p* < 0.001

*Note*: *p‐value was calculated by comparing DSC (sCT, CBCT) versus DSC (dpCT, CBCT) according to paired sample *t*‐tests.

Abbreviations: CBCT, cone‐beam computed tomography; CT; computed tomography; dpCT, deformed planning CT; DSC, dice similarity coefficient; sCT; synthetic CT.

For target areas, the CTV and PTV of cervical cancer were comparatively analyzed among three registration methods, rigid registration, deformable image registration, and ROI registration. As shown in Table 2, the ROI registration method achieved the highest DSC value of 0.867 for CTV and 0.917 for PTV, significantly higher than the rigid registration method (0.798 for CTV and 0.871 for PTV) and deformable image registration method (0.846 for CTV and 0.9 for PTV), the *p*‐values were less than 0.05.

**TABLE 2:** The accuracy of three registration methods for target areas on 25 paired CT/CT data in Dataset1(DSC).

Registration methods	CTV	PTV
Rigid registration	0.798 ± 0.070	0.871 ± 0.050
Deformable image registration	0.846 ± 0.042	0.900 ± 0.033
ROI registration	0.867 ± 0.031	0.917 ± 0.020
p1^*^	< 0.001	< 0.001
p2^*^	0.048	0.044

*Note*: *p1 was calculated by comparing rigid registration and ROI registration according to paired sample *t*‐tests. p2 was calculated by comparing deformable image registration and ROI registration according to paired sample *t*‐tests.

Abbreviations: CT, computed tomography; CTV, clinical target volume; DSC, dice similarity coefficient; PTV, planning target volume; ROI, region‐of‐interest.

The accuracy of dose calculation engine, ArcherQA, was validated with 25 patients treated on Eclipse treatment planning system, which is shown in Table 3. The gamma pass rate at 2%/2 mm was 99.16% ± 0.59%, indicating the high consistency of ArcherQA and the dose calculation algorithm in the Eclipse treatment planning system.

**TABLE 3:** Gamma pass rates of ArcherQA for 25 patients with Eclipse treatment planning system (Threshold = 10%, global 3D).

Criteria	2%/2 mm (%)	2%/3 mm (%)	3%/2 mm (%)	3%/3 mm (%)
Dose_Eclipse_ versus Dose_ArcherQA_	99.16 ± 0.59	99.48 ± 0.38	99.76 ± 0.18	99.88 ± 0.11

Table 4 showed the dosimetric outcomes of targets and organs at risk for 10 cervical patients, each patient had completed 25 fractions of clinical online ART. In our clinical practice, the V_100%_ of CTV needed at least 99%, the V_100%_ of PTV needed at least 95%, the D_99%_ of CTV, and the D_95%_ of PTV were more than the prescribed dose (45 Gy). Benefited from the plan re‐optimization, the Ethos adaptive plan could achieve better or equivalent dosimetric parameters than the original plan, while the dosimetric parameters of the Ethos scheduled plan were slightly worse than the original plan and the Ethos adaptive plan, mainly reflected in the reduction of target dose and the increase of organs’ dose. The results of our proposed ArcherQA Adaptive Check were similar to the Ethos scheduled plan.

**TABLE 4:** Dosimetric parameters of targets and organs at risk for 250 online ART fractions of 10 patients in Dataset3.

Dose parameters	Original plan	Ethos adaptive plan	Ethos scheduled plan	ArcherQA Adaptive Check
CTV_V_100%_(%)	99.88 ± 0.20	99.93 ± 0.10	96.32 ± 6.19	96.13 ± 9.73
CTV_D_99%_(Gy)	45.38 ± 0.09	45.38 ± 0.13	44.04 ± 1.66	44.31 ± 4.79
PTV_V_100%_(%)	95.79 ± 0.85	97.01 ± 0.77	89.57 ± 6.95	90.38 ± 9.88
PTV_D_95%_(Gy)	45.14 ± 0.17	45.33 ± 0.14	43.62 ± 1.27	43.90 ± 4.32
Bladder_V_40Gy_(%)	26.10 ± 6.85	22.75 ± 5.44	22.24 ± 6.05	24.60 ± 8.12
Rectum_V_40Gy_(%)	28.47 ± 11.22	34.99 ± 11.18	34.09 ± 15.37	38.87 ± 16.71
Bowel_V_40Gy_(%)	82.55 ± 5.48	82.04 ± 5.54	83.61 ± 5.95	83.39 ± 5.70
Bone Marrow_V_10Gy_(%)	2.27 ± 2.75	1.67 ± 1.90	2.01 ± 2.12	2.36 ± 2.52
Femur head left_V_30Gy_(%)	1.65 ± 2.29	0.96 ± 1.53	1.67 ± 2.20	1.89 ± 2.39
Femur head right_V_30Gy_(%)	11.15 ± 4.01	13.98 ± 4.02	14.68 ± 3.97	15.51 ± 4.03

Abbreviations: ART, adaptive radiation therapy; CTV, clinical target volume; PTV, planning target volume.

**FIGURE 4:**
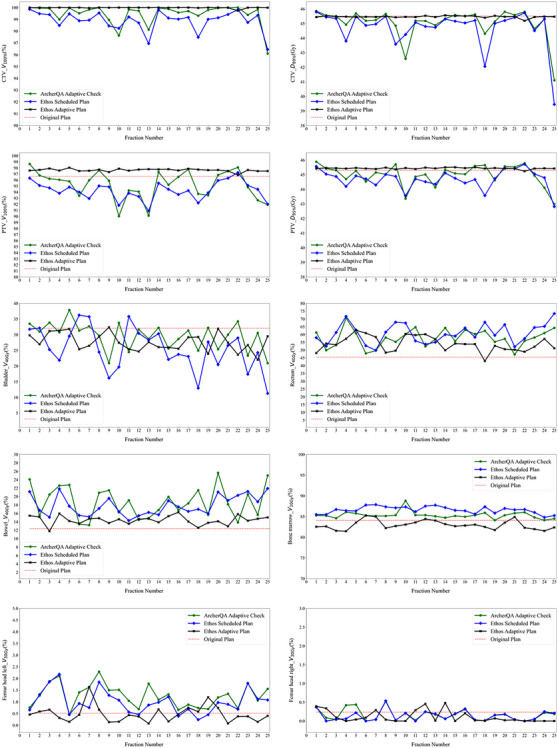
Dosimetric parameters of targets and OARs for 25 fractions of patient 9. OARs, organs at risk.

**FIGURE 5:**
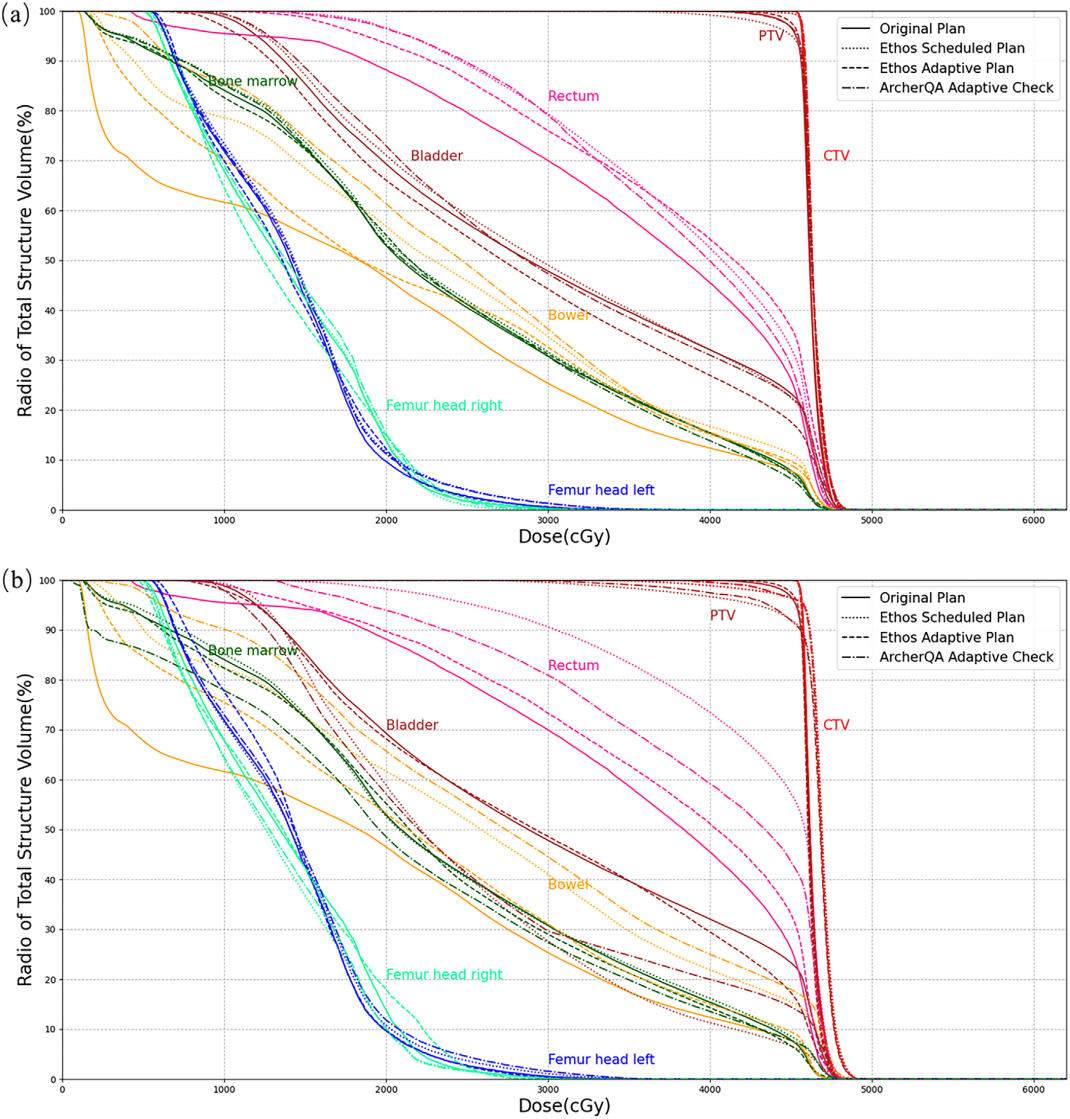
Dose volume histograms of two fractions in patient 9. (a) Fraction 2, a slight difference between the Ethos adaptive plan and Ethos scheduled plan. (b) Fraction 25, significant difference between the Ethos adaptive plan and the Ethos scheduled plan.

Although an adaptive plan could achieve better dose transmission overall, not every fraction was like this. As shown in Figure 4, the difference between the Ethos adaptive plan and the Ethos scheduled plan varied at different fractions. Take the D_99%_ of CTV as an example, the scheduled plan achieved values no worse than the adaptive plan at fractions 1, 5, 8, and 22, there were other fractions that the values of the scheduled plan slightly decreased. As a whole, there were similar results between the Ethos scheduled plan and our proposed ArcherQA Adaptive Check. Figure 5 shows the dose volume histograms of two fractions in patient 9 as examples, fraction 2 (Figure 5a) and fraction 25 (Figure 5b). In Figure 5a, there was a slight difference between the Ethos adaptive plan and the Ethos scheduled plan, which was recommended for direct irradiation without online ART. In Figure 5b, there is a significant difference between the Ethos adaptive plan and the Ethos scheduled plan, which was recommended to receive online ART to get more accuracy of dose transmission.

As for the cost time, the average time for the Ethos scheduled plan and Ethos adaptive plan was 13 and 15 min, while the ArcherQA Adaptive Check needed less than 3 min.

## 4 DISCUSSION

In the conventional radiation therapy process, tumor patients need multiple fractional exposures to complete a course of treatment. If the changes caused by anatomical structure and physiology in the patient's body are ignored during this process, radiation dose transmission may cause errors, which may ultimately lead to treatment failure. The strategy of ART is proposed to adjust the radiotherapy plan timely based on changes in the patient's anatomical structure during the treatment process. Currently, an online ART process guided by Cone Beam CT (CBCT) before fractional therapy needs 20−30 min to complete, putting pressure on already busy radiotherapy departments. In clinical practice, each execution of online ART consumes a lot of manpower and resources, and online ART is not necessary for all fractions. This study proposes a treatment plan evaluation method based on fractional CBCT images to complete a quantitative evaluation of the plan based on accurate contour information and accurate dose distribution. The key technologies used in this study include artificial intelligence‐based image registration, synthetic CT generation, and auto‐segmentation techniques, as well as GPU‐accelerated MC dose calculation technology. The entire process can be completed within 3 min automatically, and the results indicate that the process can quantify the dosimetric differences caused by anatomical structures, providing a reference for clinical ART processes.

The structures that cervical cancer patients need to be delineated during ART include the tumor target areas and its surrounding organs. Referred to the Ethos system,^37^ the structures were divided into three types: influencers, rigid organs, and target areas. Three automatic contour generation methods were adopted. Influencers refer to organ structures located around the target area that are prone to deformation and affect the target structure. In this study, the influencers are the small intestine, rectum, and bladder. Rigid organs refer to the organs at risk that are not prone to deformation, which are the femoral head, bone marrow, spinal cord, and so forth. We have demonstrated in previous studies that cascaded network registration based on artificial intelligence achieves the same accuracy as B‐spline registration while achieving higher efficiency (achieving deformable image registration process within 1 s). However, both registration methods perform poorly for easily deformable organs. Table 1 shows that the deformable registration method has a DSC of less than 0.7 for the rectum and less than 0.8 for the bladder, which cannot meet the requirements of the TG‐132 report,^29^ The DSC of the small intestine is 0.844 because the small intestine structure in this study is relatively large, and changes in anatomical structure have a small impact on the accuracy of the small intestine contour. In contrast, the accuracy of auto‐segmentation contours based on sCT is better than that propagated by deformable registration for small intestine, rectum, and bladder. Therefore, the auto‐segmentation with sCT images is used to obtain contour information of influencers, which is also better than the CBCT based auto‐segmentation in Ethos system. Although auto‐segmentation based on sCT performs well for rigid organs, there is little difference in accuracy compared to deformable registration method. The deformable image registration is more reliable to get the contours of rigid organs. After obtaining the contours of all organs, the contour information of these organs is used to guide the formation of deformable variable field for propagating the target structures (ROI registration). Table 2 demonstrates the superiority of ROI registration for CTV and PTV. Meanwhile, the input for ROI registration is the mask of organs, and the poor quality of CBCT images does not affect the accuracy of ROI registration. Therefore, ROI registration was used to automatically obtain the contour information of the target area in this study. Our proposed method provides rich tools for manually modifying contours, allowing users to adjust the contours of target areas and organs at risk when necessary.

Dose deformation by deformable vector field has many applications in ART,^38^, ^39^, ^40^ which can accumulate the dose from fractional radiotherapy into the planning CT image to comprehensively evaluate the radiotherapy dose received by patients throughout the entire treatment process. On the other hand, some researchers^14^, ^16^ used the dose deformation from the planning image to the fractional images for planning evaluation. For dose accumulation in ART, the deformation field is used to map the dose onto the planning CT image, and the accuracy of dose accumulation can only be improved by optimizing the deformation registration algorithm currently. However, dose calculation can be recalculated on fractional images for plan evaluation to obtain dose distribution based on the latest anatomical structure, which can obtain more accurate plan evaluation results. The dose calculation algorithm and corresponding electron density information are required to complete dose calculation on fractional images. The MC algorithm is the gold standard for dose calculation, but the CPU‐based calculation time is too long to promote in clinical practice. The dose calculation method used in this study is a GPU accelerated MC algorithm with high accuracy and efficiency, which can complete the dose calculation process within 1 min. The dose verification algorithm in Ethos system is the Collapsed cone convolution (Mobius3D), whose accuracy is less than MC algorithm used in this study. The image data calculated in this study are deformed CT images or synthetic CT images, which is demonstrated in our previous studies that the dose distributions based on these two images have very high consistency. The Ethos system also uses deformed CT images for dose calculation.^36^ Although iCBCT has significantly improved image quality, there are still some cavity artifacts and the accuracy of dose calculation is lower than that of deformed CT or synthetic CT images.

We have demonstrated in previous study^18^ that the error in dosimetric parameters caused by contour differences is much greater than that caused by the difference in dose distribution. In clinical practice, more attention needs to be paid to manual modification and confirmation of contour information. The algorithms in this study have been integrated into the commercial validation software ArcherQA, with convenient and fast manual contour modification tools and rich dose analysis tools. If researchers need to manually adjust the contours of the target area and organs at risk during the use of this algorithm, the contours can be adjusted quickly. The dosimetric results showed that there is excellent consistency between the Ethos scheduled plan and our proposed ArcherQA Adaptive Check. If the contours can be quickly adjusted, it will make the plan evaluation more accurate.

Some limitations should be noted in this study. First, our research is currently based on iCBCT images of Halcyon machines, whether the above process can be completed on CBCT images of conventional accelerators is the question we need to verify and solve in the next step. In addition, the process of this study is based on cervical cancer patients and the performance of other tumor patient data are also an issue we will explore in the future.

## 5 CONCLUSIONS

In this study, we proposed a quantitative plan evaluation method based on fractional CBCT images for the first time, which innovatively integrates artificial intelligence technology and GPU‐based fast MC calculation method to the ART process. The main algorithms had been integrated into the commercial software module, ArcherQA Adaptive Check. The results of testing based on 10 clinical ART patients show that this method can complete the automatic evaluation of plans based on fractional CBCT images within 3 min, reducing the error of dosimetric parameters compared with the original plan. To sum up, this study provided an effective quantitative analysis method and software tool for dosimetric differences caused by anatomical structural changes in the ART process.

## AUTHOR CONTRIBUTIONS

Paper idea: Yankui Chang, Xi Pei, Qiang Ren. The source of datasets: Yongguang Liang, Bo Yang. Model: Yankui Chang, Haotian Wu, Lingyan Li. Writing of the paper: Yankui Chang, Lipeng Jiang, Qiang Ren, Xi Pei.

## CONFLICT OF INTEREST STATEMENT

Authors Xi Pei, Qiang Ren, Haotian Wu, and Lingyan Li are employed by Anhui Wisdom Technology Co., Ltd. The remaining authors declare that the research is conducted in the absence of any commercial or financial relationships that could be construed as a potential conflict of interest.

## ETHICS STATEMENT

The studies involving human participants were reviewed and approved by the Medical Ethics Committee of Peking Union Medical College Hospital. The patients/participants provided their written informed consent to participate in this study

## Data Availability

The raw data supporting the conclusions of this article will be made available by the authors, without undue reservation.
